# Systematic Generation of Diverse Benchmarks for DNN Verification

**DOI:** 10.1007/978-3-030-53288-8_5

**Published:** 2020-06-13

**Authors:** Dong Xu, David Shriver, Matthew B. Dwyer, Sebastian Elbaum

**Affiliations:** 8 Microsoft Research Lab, Redmond, WA USA; 9 University of Southern California, Los Angeles, CA USA; University of Virginia, Charlottesville, VA 22904 USA

**Keywords:** Neural network, Verification, Benchmark, Covering array

## Abstract

The field of verification has advanced due to the interplay of theoretical development and empirical evaluation. Benchmarks play an important role in this by supporting the assessment of the state-of-the-art and comparison of alternative verification approaches. Recent years have witnessed significant developments in the verification of deep neural networks, but diverse benchmarks representing the range of verification problems in this domain do not yet exist. This paper describes a neural network verification benchmark generator, GDVB, that systematically varies aspects of problems in the benchmark that influence verifier performance. Through a series of studies, we illustrate how GDVB can assist in advancing the sub-field of neural network verification by more efficiently providing richer and less biased sets of verification problems.

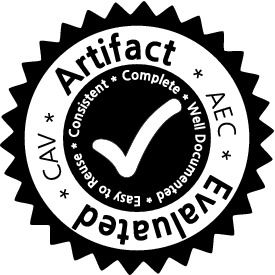



## Motivation

Advances in machine learning have enabled training of deep neural networks (DNN) that are capable of realizing complex functions that rival or exceed the performance of human-built software, e.g., [27, 32, 41]. This success has led system developers to deploy, or consider deployment of, DNN models in critical systems, e.g., [12, 39, 53]. Consequently, the verification of correctness properties of DNNs has become a key challenge to assuring autonomous systems, and the research community has risen to this challenge. In the three years since Katz et al. [30] presented ReLuplex at CAV 2017, researchers have published more than 20 DNN verification approaches supporting different properties and DNN architectures and spanning a range of algorithmic approaches [9, 13, 14, 18–20, 22, 29–31, 36, 45, 46, 50, 56, 59–63]. While DNN verification has its own unique challenges, it is also a recent example in the long-history of domain-specific verification research, e.g., for hardware [25], software [17], real-time systems [58], and cryptographic protocols [40], and can benefit from the experience of these communities.

A key lesson learned by the community is that despite the fact that verification emphasizes the development of theoretical and algorithmic techniques, *advances in verification research often arise from understanding how different algorithmic and implementation approaches compare* – a process that requires empirical study. Empirical study in verification is common, but unlike many other fields of computer science, for decades it has organized *verification tool competitions* that serve as a regular and long-running form of community-driven empirical study. Researchers tracked the progress of SMT solvers over a span of 6 years at these community-driven empirical studies and found that repeatedly “a certain solver presents a key idea that improves the performance in a particular division, and this idea is implemented by most solvers” in the following year [7]. Enabling the type of comparative studies that drive such advances requires *verification benchmarks* – a fact that the verification community has recognized for at least 25 years, e.g., [8, 10, 33, 43, 55].

Benchmarking in verification has evolved in response to the demands of empirical study within the field, e.g., [1–4], to support two objectives: (A1) *assessment of the state-of-the-art* and (A2) *comparison of alternative approaches*. In support of these, the verification community has favored benchmarks that: (R1) **are diverse in structure and difficulty**; (R2) **represent verifier use cases**; and (R3) **evolve as verification technology advances**.

The verification benchmarking and competition literature suggests that these requirements are widely accepted. For example, the TPTP benchmark’s stated goals include R1 (“contains problems varying in difficulty”), R2 (“spans a diversity of subject matters”), and R3 (“is up-to-date”, “provides a mechanism for adding new problems”) [54]. Moreover, these requirements are promoted, either explicitly or implicitly, by many of the regularly held verification competitions. To meet R1 and R2 SAT competitions construct benchmarks that include problems from six different domains: software, hardware, A.I, obstruction, combinatorial challenges, and theorem proving [4]. SAT competitions since 2017 have instituted a *bring your own benchmarks* policy that requires verifier developers to submit 20 new benchmarks with at least 10 that are “not too easy” or “too hard” – which helps to address R1 and R3. SMT competitions have used selection criteria that are biased towards these same requirements, e.g., “balancing the difficulty of benchmarks” [7].

Verification competitions have undoubtedly been a positive force for developing high-quality verification benchmarks, but prior to their existence researchers were forced to develop their own “benchmarks” – a collection of verification problems on which they evaluate their techniques and perhaps others. This is the situation that the subfield of *DNN verification* finds itself in.

The risk in letting technique developers choose their own benchmark is selection bias – that the selected problems do not represent a broad or important population of problems. For example, if an SMT benchmark were selected based on the constraints generated by symbolic execution tools they would be structurally biased, consisting only of conjunctive formula. As another example, if a SAT benchmark were generated randomly it is likely that a large portion of the benchmark would not represent realistic use cases.

Good benchmarks are expensive to develop, e.g., [11], but they are an invaluable resource for advancing a research community. When well designed they seek to balance requirements R1-R3 and to support a fair and accurate assessment of the state-of-the-art and comparison between alternative algorithmic and implementation approaches. This paper reports on GDVB, the first *framework for systematic* ***G****eneration of* ***D****NN* ***V****erification problem* ***B****enchmarks*, that meets the de-facto requirements for verification benchmarks, R1–R3, in order to support objectives A1–A2 for the rapidly evolving field of DNN verification.

GDVB takes a **generative** approach to benchmark development – an approach that has risen in popularity in recent years [5, 35, 64]. Unlike, other generative benchmark approaches GDVB seeks to systematically cover variations in verification problems that are known to influence verifier performance. Towards that end, GDVB is parameterized by: (1) a set of *factors* known to influence the performance of DNN verifiers; (2) a *coverage* goal that determines the combination of factors that should be reflected in the benchmark; and (3) a *seed* verification problem from which a set of variant problems are generated. From these parameters, it computes a constrained mixed-level covering array [15] defining a set of factor-value tuples. Each tuple defines how the seed verification problem can be transformed to give rise to a verification problem capable of exposing performance variation in a DNN verifier.

As a benchmark generator GDVB naturally meets requirement R3. By starting from a seed network representing a DNN verification use case, GDVB is guaranteed to meet R2. As we discuss in Sect. 4, the use of factors allows GDVB to produce systematically diverse verification problems both in terms of structure and difficulty in order to meet requirement R1. Moreover, GDVB offers the potential to reduce selection bias in performing evaluations of DNN verifiers, since it assures coverage of a space of performance related factors. Finally, GDVB is designed to support the rapidly evolving field of DNN verifiers by allowing the generation of benchmarks, e.g., from new seeds as verifiers improve, as new performance factors are identified, and to target challenge problems in different DNN domains, e.g., regression models for autonomous UAV navigation [39, 53].

The contributions of this paper are: identification of the need for unbiased and diverse benchmarks for DNN verification; a study of factors that affect the performance of DNN verification tools (Sect. 3); the specification of a verification benchmark as the solution to a constrained mixed-level covering array problem (Sect. 4); the GDVB algorithm for computing a benchmark from a verification problem by transforming the neural network and correctness specification (Sect. 4.3); the evaluation of GDVB on multiple state-of-the-art DNN verifiers using different seed verification problems that demonstrates how GDVB results can support the evaluation of DNN verifiers (Sect. 5); and the GDVB tool.

## Background and Related Wok

**Deep Neural Networks (DNN).** A DNN is trained to accurately approximate a target function, $f: \mathbb {R}^{d} \rightarrow \mathbb {R}^{r}$. A network, $n: \mathbb {R}^{d} \rightarrow \mathbb {R}^{r}$, is comprised of a graph of *L* hidden layers, $l_1, \ldots , l_L$, along with an input layer, $l_{in} = l_0$, and output layer, $l_{out} = l_{L+1}$. Each hidden layer defines an independent function, where their composition when applied to the output of $l_{in}$ generates values in $l_{out}$ that define the network output.

Hidden layers are, generally, comprised of a set of *neurons* that accumulate a weighted sum of their inputs from the prior layer and then apply an *activation function* to determine how to non-linearly scale that sum to compute the output from the layer. A variety of different activation functions have been explored in the literature, including: rectified linear units (ReLU), sigmoid, and tanh.

The design of a DNN involves choosing an appropriate set of *layer type*s, e.g., convolutional, maxpooling, fully-connected, the instantiation of those layers, e.g., the number of neurons, the specific activation function, and the definition of how layers are interconnected. Together these comprise the DNN *architecture* [23].

Networks are trained using a variety of algorithmic strategies with the goal of minimizing the loss in the approximation of the learned function relative to some proxy for *f*, e.g., labeled training data. The training process is stochastic, e.g., initial weight values are randomized, which leads to variation in *n* even when architecture, training algorithm, and training data are fixed.

Section 3 reveals how DNN architecture can influence verification performance.

**DNN Specifications.** Given a network $n: \mathbb {R}^{d} \rightarrow \mathbb {R}^{r}$, a property, $\phi$, defines a set of constraints over the inputs, $\phi _{{\textit{\textbf{x}}}}$, and an associated set of constraints over the outputs, $\phi _{y}$. Verification of *n* seeks to prove: $\forall {{\textit{\textbf{x}}}\in \mathbb {R}^d}: \phi _{{\textit{\textbf{x}}}}({\textit{\textbf{x}}}) \Rightarrow \phi _{y}(\mathbf {N}(x))$ where $\mathbf {N}(x)$ is running the neural network *n* with input *x*.

Specifying behavioral properties of DNNs is challenging and is an active area of research [24]. In [30], a set of 188 purely conjunctive properties, of the form described above, were defined for a simple neural network, with 7 inputs, encoding of a rule set for autonomous aircraft collision avoidance (ACAS). In [44, 59, 60], properties expressing output range invariants were used, for example, that the steering angle never exceeded an absolute value of 30$^\circ$. Much of the work on DNN verification has focused on local robustness properties [50–52], which state that for a selected target input the output of the network is invariant for other inputs within a specified distance of the target.

Section 3 reveals how the specification can influence verification performance.

**DNN Verification Methods and Tools.** There are a variety of different algorithmic and implementation approaches taken to verifying the validity of a DNN with respect to a stated correctness property.

### Definition 1

A DNN verification problem, $\langle n, \phi \rangle$, is comprised of a DNN, *n*, and a property specification, $\phi$. The outcome of a verification problem for a DNN verifier indicates whether $n \models \phi$ is valid, invalid, or unknown – indicating that the problem cannot be determined to be either valid or invalid.

A recent DNN verification survey [37], classifies approaches as being based on reachability, optimization, and search algorithms – or their combination. Reachability methods begin with a symbolic encoding of an input set and compute, for each layer, a symbolic encoding of the output set. They vary in the symbolic encodings used, e.g., intervals, polyhedra, and in the degree of overapproximation they introduce [22, 46, 50, 63]. Optimization methods formulate verification as an optimization problem whose solution implies the validity of $\phi$ [9, 19, 38, 45, 56, 62]. Search methods work in combination with reachability and optimization, by decomposing the input space to formulate verification sub-problems that are discharged by the above techniques [13, 14, 18, 20, 29, 30, 59–61].

In this paper, we use implementations of the following verifiers: ERAN [50], BaB [14], Neurify [59], Planet [20], and ReLuplex [30].

**Verification Benchmarking.** We covered the broad landscape of work on benchmark development for verification in (Sect. 1). There have been efforts to develop benchmarks within a variety of different verification problem domains, e.g. hardware [25], software [17], real-time systems [58], cryptographic protocols [40], and for different encodings of verification problems, e.g., model checking [33], SAT [4], SMT [8], and theorem proving [55].

In recent work on DNN verification, researchers have shared collections of examples that, in a sense, serve as informal benchmarks and permit comparative evaluation, e.g. [30, 50]. While valuable, these examples were not intended to, and do not, comprise a benchmark meeting requirements R1–R3. To our knowledge, GDVB is the first approach to achieving those goals for DNN verification.

For several years, the SAT community has been exploring scalable benchmarks, e.g., [21, 35]. For instance, to explore conflict-driven clause learning (CDCL) SAT solver performance, Elffers et al. [21] used crafted parameterized benchmarks that can be scaled with respect to different factors that may influence performance. We conduct a similar domain analysis of factors, but focus on the landscape of DNN verification algorithms developed to date. Like this line of work, GDVB advocates a scalable approach to benchmark generation. As described in Sect. 4, GDVB starts with seed problems that are challenging for current verifiers and “scales them down”, but it can also be applied to start with easier seed problems and “scale them up” as more typical of the prior work on scalable benchmarking.

**Verification Benchmark Ranking.** The verification community has explored a variety of ranking schemes for assessing the cost-effectiveness of techniques. A key challenge is that verification techniques vary not only in their cost, e.g., time to produce a verification result, but also in their accuracy, e.g., whether they produce an *unknown* result. For example, SAT competitions have employed a range of scoring models, e.g., purse-based ranking, *solution-count ranking* (SCR), careful ranking, and penalized average runtime (PAR2) [6]. SCR, which counts the number of solved problem instances and uses verification time as a tie breaker [57], is the scoring system of choice [1, 4]. In Sect. 5, we report DNN verifier performance using both SCR and PAR2 scoring systems.

**Covering Arrays.** In Sect. 3 we explore factors that influence DNN verifier performance. Studying all their combinations would be cost prohibitive, so we consider weaker notions of coverage.

A covering array defines a systematic method for testing how combinations of parameter values influence system performance [16]. A covering array is an $N \times k$ array. The *k* columns represent *factors* that may influence performance and cells can take on *v* *levels* – defining settings for factors. The *N* rows of the array define combinations of factor-levels. Arrays are defined to achieve a *strength* of the coverage, *t*. $t=2$ defines pairwise strength, which means that all pairs of levels for all factors are present in some row of the covering array.

We require a richer form of covering array that permits the number of levels to vary with different factors, i.e., a mixed-level covering array (MCA), and that can constrain specified factor-level combinations, e.g., by forbidding their inclusion in the MCA. By modeling each factor as a variable and its levels as the domain of the variable, one can express constraints as propositional logic formulae over equality terms; if the levels are ordered then richer underlying theories can be applied. A constrained-MCA defines an MCA that is consistent with a given constraint, *C*.

### Definition 2

Constrained Mixed-level Covering Array (Definition 2.9 from [15])

$CMCA(N; t, k, (|v_1|, |v_2|,..., |v_k|),C)$ is an $N \times k$ array on |*v*| symbols, where $|v| = \sum _{i=0}^{k}{|v_i|}$, with the following properties: 1) Each column $i(1 \le i \le k)$ contains only elements from a set $S_i$ of size $|v_i|$, 2) the rows of each $N \times t$ subarray cover all *t*-tuples of values from the *t* columns at least one time, and 3) all rows are models of *C*.

**Transforming Neural Networks.** The GDVB approach manipulates factors that influence DNN verifier performance to construct a diverse benchmark. For DNN construction, we leverage a recent approach, R4V [47], that given an original DNN and an architectural specification automates the transformation of the DNN and uses distillation [28] to train it to closely match the test accuracy of the original DNN. R4V transformation specifications can be written to change a number of architectural parameters of a network including: the input dimension, the range of values for each input dimension, the number of layers, the number of neurons per layer, the number of convolutional kernels, and the stride and padding of a convolutional layer.

## Identifying Factors that Influence Verifier Performance

As discussed in Sect. 1 the verification community has acted to create policies that incentivize *diverse* benchmarks. Diversity is desirable in a benchmark because it (a) demonstrates the range of applicability of a verification technology and (b) exposes performance variation within and across verification technologies. Consider, that the SMT competition benchmark selection process seeks to “include equal numbers of satisfiable and unsatisfiable benchmarks at different levels of difficulty” [7]. This is due to the fact that the SMT community understands that the satisfiability or unsatisfiability of a benchmark problem is a factor that influences verifier performance^1^.

GDVB seeks to make factors influencing verifier performance explicit and to manipulate them to generate a diverse benchmark. To determine an initial set of factors for DNN verifiers we began with an analysis of the literature, which identified several candidate factors, and then conducted a targeted and exploratory **factor study** to identify whether *manipulating a factor could influence some performance measure of some DNN verifier*. This study only aims to identify such factors and does not seek to characterize the complex relationship between factors and DNN verifier performance; for example, we do not aim to capture a comprehensive set of factors, assess the independence of or relations between factors, or rank factors in terms of their degree of influence. A richer and more detailed factor study might further improve the utility of GDVB, but we leave such a study to future work.

### Potential Factors

Relatively few published papers on DNN verification explicitly discuss the factors that influence performance, but nearly all of them present metrics on the verification problems they solved.

Evaluation results for ReLuplex present data on verifier outcome and solve time for local robustness properties that vary in the input center point and radius [30]; most subsequent papers report similar property variation. Evaluation results for RobustVerifier present a study of varying the number of layers in the DNN and its impact on verifier performance [36]. Evaluation results for ERAN present performance variations across a range of networks varying in the number of layers, layer types, and neurons [22, 50–52]. Bunel et al. [14] were the first that we are aware of to explicitly vary factors of DNN verification problems. They found that the performance varied with input dimension, number of neurons per layer, and number of layers across a set of 6 different DNN verifiers. All of the other papers published on DNN verification in recent years have used verification problems that varied, in an ad-hoc fashion, over a subset of the above factors.

### Exploratory Factor Study

As in other verification domains, DNN verifier performance is multi-faceted. In our study, we consider both verification time and accuracy. We say that the result of a verification problem is *accurate* if a verifier determines conclusively that the problem is *valid* or *invalid*, result as opposed to *unknown*^2^.

**Fig. 1.**
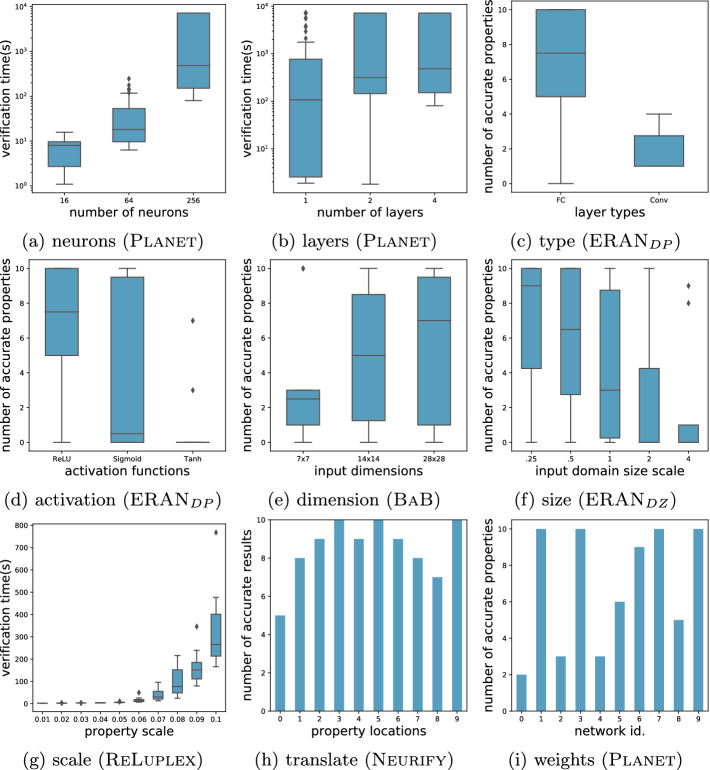
DNN verifier performance across factors

We study factors associated with both properties and DNNs. Based on the literature analysis, we identified 2 factors related to the correctness property: *scale* and *translation*. Scaling a property involves increasing the size of the input domain which will involve *more DNN behavior* in verification. Translating a property involves moving it to a different location in the input domain which will involve *different DNN behavior* in verification. For robustness properties, scaling and translation involve changing the radius and center point of the hypercube describing the input space under verification. One might wonder whether rotation of a property can influence verification performance. For robustness properties, this seems unlikely given their symmetry, but it could be a factor for more irregular input regions – we leave this for future work.

Based on the literature analysis, we identified 4 factors related to the DNN: number of *neurons*, number of *layers*, the *type* of layers, the input *dimension*. We conjectured that an additional 3 factors might impact verifier performance: the type of *activation* function, the input domain *size*, and the learned *weights*.

Our exploratory factor study is opportunistic in that we seek to find a verification problem for which manipulation of a selected factor exhibits performance variation. Towards this end, we conducted a series of trials where we vary a factor hypothesized to influence verification performance, while holding all other factors constant and report the results in Fig. 1. We studied variations of networks for the MNIST task and considered local robustness properties since these were well-supported across a range of different verifiers. We used different verifiers across the study: ReLuplex, Planet, Neurify, BaB, ERAN with the DeepPoly (DP) and DeepZono (DZ) abstract domains. We now briefly describe the trials and then summarize the outcome.

- **Number of Neurons:** The architecture of the DNN was fixed, with 4 fully-connected layers using ReLU activation functions, and the total number of neurons was varied (16, 64, 256) – they were spread evenly across layers. Each network is trained 10 times and verified on 100 local robustness properties. Figure 1(a) plots the number of neurons versus verification time for Planet. **Verification time can increase with the number of neurons.**
- **Number of Layers:** We use the same context as for the neuron factor study, except that we fixed the number of neurons at 256 and vary the number of layers (1, 2, 4). Figure 1(b) plots the number of layers versus verification time for Planet. **Verification time can increase with the number of layers.**
- **Layer Types:** We use a pair of two-layer neural networks, with the same number of neurons, where one has a fully-connected layer and the other a convolutional layer. Each network is trained 10 times and verified on 10 local robustness properties. Figure 1(c) plots layer type versus the number of properties for which accurate results are produced using $\textsc {ERAN} _{DP}$. **Verification accuracy can vary with layer type.**
- **Activation Function:** We use the fully-connected network from the layer types study, we generated three networks by altering the activation function to use sigmoid and tanh. The training setup and properties remain the same as in the previous trial. Figure 1(d) plots the activation function versus the number of properties for which accurate results are produced using $\textsc {ERAN} _{DP}$. **Verification accuracy can vary with activation function.**
- **Input Dimension:** We use 3 architectures that differ only in their input dimension which is scaled ($\frac{1}{16},\frac{1}{4},1$) relative on the original problem. The training setup and properties are from the layer type study. Figure 1(e) plots the input dimension versus the number of properties for which accurate results are produced using BaB. **Verification accuracy can increase with increasing input dimension.**
- **Input Size:** We use 5 architectures that differ only in the range of values of their inputs which are scaled (${\frac{1}{4},\frac{1}{2},1,2,4}$) based on the original problem. The training setup and properties are from the layer type study. Figure 1(f) plots the input size versus the number of properties for which accurate results are produced using $\textsc {ERAN} _{DZ}$. **Verification accuracy can decrease with increasing input domain size.**
- **Property Scale:** We use a single-layer network and reuse the training setup and properties from the layer type study. We scale the properties ($0.01 - 0.1$) to generate verification problems. Figure 1(g) plots property scaling versus the verification time using ReLuplex. **Verification time can increase with increasing property scale.**
- **Property Translation:** We replicated the property scale study, but held the scale fixed and translated the center point of the local robustness property to 10 other locations. Figure 1(h) plots the number of DNNs for each of the 10 translated properties for which accurate results could be produced using Neurify. **Verification accuracy can vary with property translation.**
- **Network Weights:** Building of the property studies, we explore the verification of 10 scaled property variants across the same network trained 10 times with different initial weights. Figure 1(i) plots the number of accurate properties for which the results could be produced using Planet. **Verification accuracy can vary with the learned weights of the network.**

**Exploraty Study Findings.** Varying the factors studied influences the performance of different DNN verifiers differently – in terms of time or accuracy. For example, we found that: varying input dimension impacts BaB ’s accuracy, but not ReLuplex ’s; varying input domain size impacts $\textsc {ERAN} _{DZ}$’s accuracy, but not Neurify ’s; and varying property scale impacts ReLuplex ’s verification time, but not Neurify ’s.

This study provides a starting set of viable factors that can be used to parameterize the GDVB approach to produce verification problem benchmarks in which those factors are systematically varied. Futhermore, as we discuss in Sect. 4, GDVB generative process allows for us to accommodate information about new factors that might be revealed in future factor studies.

## The GDVB Approach

The goal of GDVB is to meet requirements R1–R3 by producing a *factor diverse* benchmark that (a) reflects aspects of the complexity encoded in a real verification problem that acts as a seed for generation $\langle n_s, \phi _s \rangle$, (b) varies aspects of the problem that are related to verifier performance, (c) accounts for interactions among those factors, and (d) is comprised only of well-defined verification problems.

Rather than synthesize random verification problems, we seed the generation process in order to generate a benchmark that reflects the complexity of the seed problem. This permits benchmarks to be generated to reflect the challenges present in different DNN problem sub-domains.

Factors, like those described in Sect. 3, may interact; changes to one factor may mask or amplify DNN verifier performance changes arising from another. Exploring all combinations of factors is expensive, but by using covering arrays we can systematically explore interactions among factors. Accounting for such interactions helps to produce a benchmark that is *less biased* than one that only covers individual factor variations.

Not all combinations of factors are possible. For example, if one reduces the number of layers in a network to 0, then it is not possible to preserve the number of neurons in the original network. Thus, benchmark generation must take into account constraints among factors to ensure that only well-defined problems are included in a benchmark.

### Factor Diverse Benchmarks

Consider a set of factors, *F*, with a set of levels, $L_{f}$, for each factor, $f \in F$; we refer to $L_{f}$ as the *level set* of *f*. For a verification problem, *p*, let *l*(*p*) be the set of factor levels corresponding to the problem. A benchmark, *B*, is a set of verification problems and we can denote the factor levels for the benchmark as $l(B) = \{ l(p) \mid p \in B \}$.

The simplest form of diversity for a benchmark is requiring that all individual factor levels be present in at least one verification problem, $\forall f \in F : \forall l \in L_f : \exists p \in l(B) : l \in p$. However, this diversity fails to account for interactions among factors. The simplest form of interaction-sensitive diversity considers pairs of factors, but as we discuss below our approach generalizes to any arity of factor-level coverage.

For a pair of factors, $f, f' \in F$, the Cartesian product of their level sets defines the set of all pairwise combinations of their levels. Across all factors the set of such pairs is $pairs(F) = \{ (l,l') \mid f, f' \in F \wedge f \not = f' \wedge l \in L_f \wedge l' \in L_{f'} \}$. A *pairwise diverse benchmark* is one in which

$$ \forall (x,y) \in pairs(F) : \exists p \in l(B) : (x,y) \in \{ (x',y') \mid x' \in p \wedge y' \in p\} $$

Constraints on allowable combinations of factors serve to restrict a benchmark. A pairwise exclusion constraint, $\gamma (F) \subseteq pairs(F)$, requires that

$$ \forall (x,y) \in \gamma (F) : \forall p \in l(B) : \lnot (x \in p \wedge y \in p) $$

We write $\gamma$ when *F* is understood from the context.

The arity of factor-level coverage and exclusion constraints can vary independently. It is common for factor-level coverage to be uniform and to generalize it to *t*-way coverage, i.e., to require coverage of the elements of the Cartesian product of the level sets of *t* factors. On the other hand, as observed in prior work [15], constraints generally involve a mix of arity. To denote this generality we define $\varGamma \subseteq \bigcup _i \gamma _i$ where $\gamma _i$ defines the set of possible *i*-way exclusion constraints.

#### Example

Consider the DAVE-2 DNN which accepts 100 by 100 color images and infers an output indicating the steering angle [12]. DAVE-2 is comprised of 5 convolutional layers with 55296, 17424, 3888, 3136, and 1600 neurons, respectively, followed by 4 fully connected layers with 1164, 100, 50, and 10 neurons, respectively. All 82668 neurons use ReLU activations. One can define a local robustness property for DAVE-2 as

$$ \phi = \forall {\textit{\textbf{x}}}\in i \pm 0.02 : \Vert \textsc {DAVE-2} ({\textit{\textbf{x}}}) - \textsc {DAVE-2} (i) \Vert \le 5 $$

which states that for a given an input image, *i*, all inputs within a distance of 0.02 will result in an inferred steering angle within 5$^\circ$ of the angle for *i*. These yield the verification problem $\langle \textsc {DAVE-2}, \phi \rangle$.

Consider factors for the number of neurons, number of convolutional layers, and number of fully-connected layers; a tuple $(\# neuron,\# conv,\# fc)$ represents levels for these factors. For each factor consider two percentage levels: 100% and 50%. A neuron factor level of 50% indicates that a version of DAVE-2 with 41334 neurons is required. In the absence of constraints, an example pairwise factor diverse benchmark for $\langle \textsc {DAVE-2}, \phi \rangle$ consists of the following four verification problems: $(100\%, 100\%, 100\%)$, $(100\%, 50\%, 50\%)$, $(50\%, 100\%, 50\%)$, and $(50\%, 50\%, 100\%)$. The property $\phi$ is constant across the benchmark.

### From Factor Covering Arrays to Verification Problems

Given a set of factors, $F = \{f_1, f_2, \ldots , f_{|F |}\}$, and levels, $L_{f_i}$, a *t*-way factor diverse benchmark of *k* verification problems is specified by

$$ CMCA(|F |; t, k, (|L_{f_1} |, |L_{f_2} |, \ldots , |L_{f_{|F |}} |), \varGamma ) $$

Each element in this mixed level covering array specifies how to construct a verification problem in the benchmark from the seed problem.

Levels are operationalized as transformations on verification problems. We assume a sufficient set of transformations, $\varDelta$, such that a verification problem can be transformed into a form that achieves any level of any factor

$$ \forall f \in F : \forall l_f \in L_f : \exists \delta \in \varDelta : l_f \in l(\delta (\langle n_s,\phi _s \rangle )) $$

The definition of $\varDelta$ and $L_i$ must be coordinated to achieve this property.

A per-factor transformation $\delta \in \varDelta$ may impact a single component of a verification problem, e.g., reducing the number of neurons in a DNN does not impact the property, or both components, e.g., the input dimension impacts the DNN and the property by transforming the input data domain. The set of all transformations $\varDelta$ defines the set of verification problems that can be produced by application of a set of per-factor transformations to the seed problem,

$$ \varDelta (\langle n_s, \phi _s \rangle ) = \{ \langle n, \phi \rangle \mid \langle n, \phi \rangle = \delta _{f_1} \circ \delta _{f_2} \ldots \circ \delta _{f_{|F |}}(\langle n_s, \phi _s \rangle ) \wedge \delta _i \in \varDelta \} $$

The set of all possible factor level combinations is $\Pi _{f \in F} L_f$, i.e., the product of all of the per-factor levels. The set of t-way factor level combinations is

$$ c_t = \{ c | a \in \Pi _{f \in F} L_f \wedge c \subseteq a \wedge |c |= t\} $$

allowing for the interpretation of $|F |$-tuples as sets.

#### Definition 3

Given a set of factors *F*, with associated factor levels $L_f$, a *t*-way factor diverse benchmark, *B*, for a seed problem $\langle n_s, \phi _s \rangle$ with exclusion constraints $\varGamma$ is defined by the following: (1) $B \subseteq \varDelta (\langle n_s, \phi _s \rangle )$; (2) $\forall {\langle n, \phi \rangle \in B} : \forall \gamma \in \varGamma : \gamma \not \subseteq l(\langle n, \phi \rangle )$; and (3) $\forall {c \in c_t - \varGamma } : \exists {\langle n, \phi \rangle \in B} : c \subseteq l(\langle n, \phi \rangle )$

### Generating Benchmarks

GDVB is defined in Algorithm 1. We use existing techniques, e.g. Automated Combinatorial Testing for Software (ACTS) [34], for generating a CMCA for constraints specified as logical formulae where factors are variables and levels are values for those variables. A CMCA is a set of *k*-tuples. Each such tuple defines the target level for each factor for a problem in the generated benchmark. Those levels are used to transform the given seed verification problem and the resultant problem is accumulated in the benchmark.
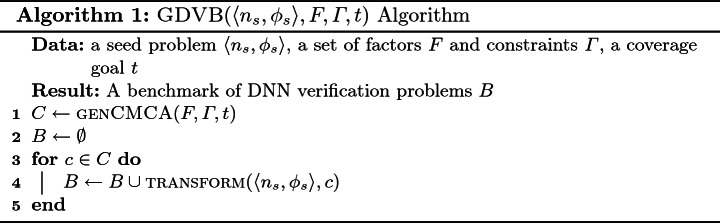


transform uses different approaches to transform the seed DNN and the property. DNN transformation builds on an approach called R4V that automates architectural transformations to DNNs by scaling (1) the number of neurons in a fully connected layer, (2) the number of kernels in a convolutional layer, (3) the input dimension, or (4) the range of values within an input dimension [47]. The first 3 of these require changes to the structure of the DNN and the last two require changes to the training data, e.g., reshaping, renormalizing. R4V ensures that the network is well-defined after transformation. transform maps factor-levels to per-layer scale parameters for R4V.

R4V permits the training of a network using network distillation which we find advantageous for GDVB because: it accelerates the training process, and it drives training to match the accuracy of the problem DNN to that of $n_s$, which reduces variation in accuracy across *B*. We adapt R4V so that after each training epoch, the learned DNN weights and the validation accuracy is recorded. When training finishes, we select the weights associated with the highest validation accuracy. Training is performed using the training data and hyperparameters for $n_s$.

Whereas R4V can be used to directly manipulate DNN architecture related factors, it can only indirectly affect the learned weights. To address this, we adopt the approach taken throughout the machine learning literature – train a network on multiple initial seeds and report performance across seeds. Thus, each DNN in *B* is trained multiple times, thereby producing a benchmark comprised of $s * |B |$ verification problems, where is the desired number of seeds.

**DNN Transformation Example.** Consider this element of the CMCA described above: $\langle (50\%, 100\%, 50\%), \phi \rangle$, applied to DAVE-2. transform would compute that 50% of the fully connected layers should be present in the resultant DNN and randomly select 2 of the 4 layers to scale by 0. The fully-connected layers are chosen at random, since the layer count factor does not consider layer ordering. If we consider the case where the layers with 100 and 50 neurons are dropped, this will eliminate 150 neurons. The other transformation required is to reduce the number of neurons by half. To do that all remaining layers will be scaled by $\frac{82668\,*\,0.5\,-\,150}{82688} = 0.498$.

**Fig. 2.**
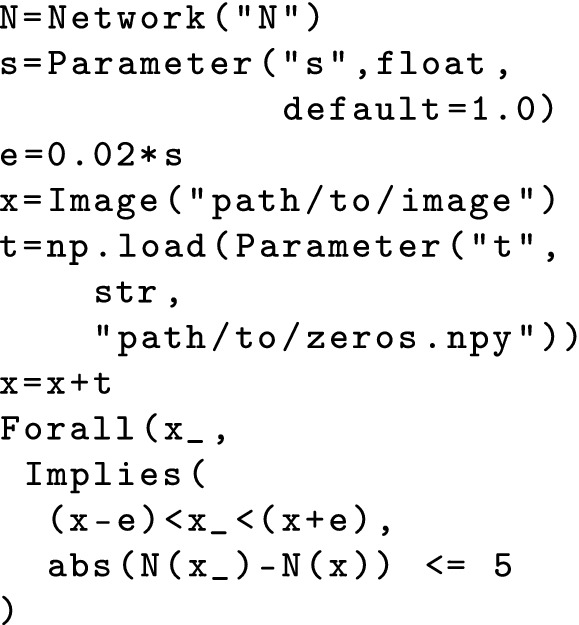
Parametric property $$\phi $$

Property transformation builds on a domain-specific language (DSL) for specifying DNN correctness properties defined by the *deep neural network verification framework* (DNNV) [48]. Specifications in this Python-based DSL are parametric and transform maps factor-levels to those parameters. For example, Fig. 2 defines the parametric local robustness property $\phi$ that is centered at the image stored at “path/to/image”, has radius 0.02, and can be translated and scaled through parameters t and s, respectively.

Restricting factors to levels that are supported by transform and using CMCA algorithms that meet Definition 2 ensures that GDVB produces a solution that meets Definition 3.

### An Instantiation of GDVB

We developed an instance of GDVB^3^ that supports a set of factors informed by the results of the study in Sect. 3, *percentage-based levels* for those factors, and a set of constraints that restrict benchmark problems to those that are non-trivial and that can be efficiently trained.

Our instantiation of GDVB supports the following factors: the total number of neurons in the DNN (**neu**), the number of fully-connected layers (**fc**), the number of convolutional layers (**conv**), the dimension of the DNN input (**idm**), the size of each DNN input dimension (**ids**), the scale of the property (**scl**), and the translation of the property (**trn**). We do not support an activation function factor because only ERAN support non-ReLU activations and, thus, using them would render other verifiers inapplicable for large portions generated benchmarks.

We use quintile factor levels, {20%, 40%, 60%, 80%, 100%}, for factors neu, idm, ids, and scl. To permit the elimination of layer types we extend these levels with an additional quintile, 0%, for fc and conv. For trn, we select a set of five translations that shift the property to be centered on a different instance of the training data; unlike the above levels this level is unordered.

Our instantiation of GDVB exclusion constraints for DAVE-2 are as follows: (1) $fc = 0 \wedge conv = 0$, (2) $conv = 0 \wedge neu \ge 20$, (3) $conv = 0 \wedge idm \ge 80$, and (4) $conv = 100 \wedge idm = 20$. The first of these requires that some layer be present. The second and third are related to the blowup in the size of fully-connected layers that results from dropping all convolutional layers which makes training difficult; limiting the total number of neurons and the reduction input dimension mitigates this. The fourth constraint ensures that the input dimension reduction results in a meaningful network; without it the dimensionality reduction achieved by sequences of convolutional layers yields an invalid network, i.e., the input to some layer is smaller than the kernel size.

These constraints were developed iteratively based on feedback from the R4V tool, which reports when transform has specified an invalid DNN, and when training failed to closely approximate the accuracy of the seed network.

We note that this instance of GDVB is flexible in that it permits the customization of levels, as we demonstrate in the next section, to generate a benchmark that focuses on variation in a subset of factors. More generally, GDVB can easily be extended to support additional factors and levels for which an instance of transform can be defined. We expect that GDVB will evolve in this way as studies of DNN verifiers are performed.

## GDVB in Use

In this section we showcase the potential uses of GDVB across a series of artifacts and verifiers, while highlighting the challenges it helps to systematically address.

### Setup

Our evaluation applies GDVB to two seed networks: $\textsc {MNIST}_{ConvBig}$ and DAVE-2. We selected $\textsc {MNIST}_{ConvBig}$ because it is one of the largest networks in ERAN ’s evaluation [50]; it includes 4 convolutional layers and 3 fully connected layers with 48,074 neurons and 1,974,762 parameters. We selected DAVE-2 to illustrate the application of GDVB to a larger network that has been the subject of other DNN analysis [42]; it has 5 convolutional layers and 5 fully connected layers with 82,669 neurons and 2,116,983 parameters.

**Table 1.** Verifiers used in GDVB study

Verifier	Algorithm
ReLuplex [30]	Search-optimization
Planet [20]	Search-optimization
BaB [14]	Search-optimization
BaBSB [14]	Search-optimization
Neurify$^\mathrm{a}$ [59]	Optimization
$\textsc {ERAN} _{DZ}$ [50]	Reachability
$\textsc {ERAN} _{DP}$ [51]	Reachability
$\textsc {ERAN} _{RZ}$ [52]	Reachability
$\textsc {ERAN} _{RP}$ [49]	Reachability

$^\mathrm{a}$We use the version of Neurify provided in DNNV [48], which is modified to be applicable to a wide range of problems, whereas the original version was hard-coded to a particular verification problem [59].

Table 1 lists the 9 verifiers we selected for our study. This list includes the most well-known verifiers and verification algorithms. We also select variations of some verification approaches. We use Branch-and-Bound (BaB), as well as a variation of Branch-and-Bound with Smart-Branching (BaBSB). Additionally, we evaluate the ERAN verifier with 4 available abstract domains: DeepZono ($\textsc {ERAN} _{DZ}$), DeepPoly ($\textsc {ERAN} _{DP}$), RefineZono ($\textsc {ERAN} _{RZ}$), and RefinePoly ($\textsc {ERAN} _{RP}$).

To evaluate verifier performance, we use the *solution-count ranking* (SCR) [57], which counts the number of properties that returned accurate verification results. Additionally, we measured the *penalized average runtime* (PAR2) [6], which is computed as the sum of the verification times for *sat* and *unsat* results and twice time limit for all other verification results.

**Table 2.**
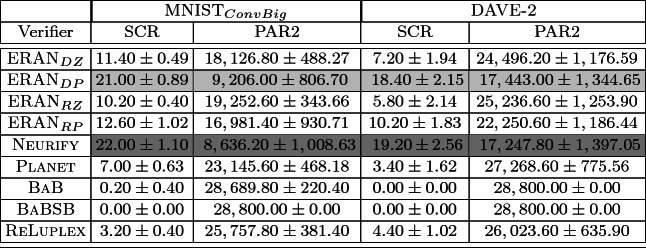
Mean & variance of SCR and PAR2 scores across benchmarks. (The darker and lighter gray boxes indicate the best and second best results.)

All training and verification took place under CentOS Linux 7. R4V transformation and distillation jobs ran on NVIDIA 1080Ti GPUs. Verification jobs were limited to 4 h and ran on 2.3 GHz and 2.2 GHz Xeon processors with 64 GB of memory, for DAVE-2 and $\textsc {MNIST}_{ConvBig}$, respectively.

### Comparing Verifiers Across a Range of Challenges

Consider the use case where a researcher is attempting to compare a new verifier (e.g., a new algorithm, a revised implementation, an extension to an existing approach) against existing verifiers. As shown earlier, for such comparison to be meaningful, many factors must be considered and properly explored. Given a seed network, a property, a set of factors, and a coverage goal, GDVB can generate a benchmark that helps to reduce bias in conducting such an evaluation.

For this use case we consider seed networks and local robustness properties similar to those from the $\textsc {ERAN} _{DZ}$ study [50] for the $\textsc {MNIST}_{ConvBig}$ verification problem and local robustness properties based on those from the Neurify study [59] for the DAVE-2 verification problem. We run an instance of GDVB using the factors and levels described in Sect. 4.4, a coverage strength of 2, and train 5 versions of each network to account for stochastic weight variation. The total time to generate and train GDVB ($\textsc {MNIST}_{ConvBig}$, ...) was 24.3 h and the resulting 30 verification problems took 401.8 h to run across all 9 verifiers. For GDVB (DAVE-2, ...) 44 verification problems were generated with training and verification times of 158.2 h and 772.4 h, respectively. CMCA generation took less than a minute for both problems. Each problem in the benchmark must be trained and verified in sequence, but across problems they can be parallelized. We exploited this to reduce the cost of running the benchmarks to 4.9 h for $\textsc {MNIST}_{ConvBig}$ and 7.9 h for DAVE-2. We measured the SCR and PAR2 score for the nine verifiers across the benchmarks.

**Fig. 3.**
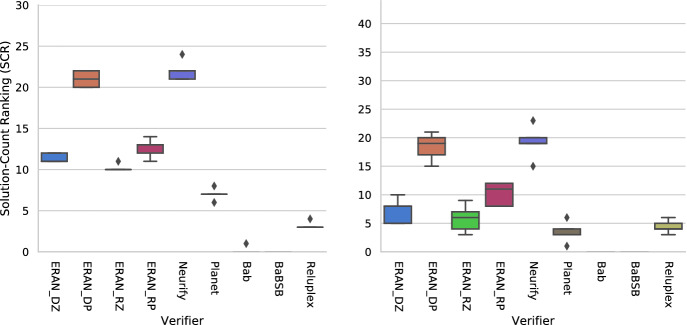
SCR score for nine verifiers on GDVB benchmarks with $\textsc {MNIST}_{ConvBig}$ (left) and DAVE-2 (right) seeds

The results are shown in Table 2. Since the SCR and PAR2 score trends are the same we depict just SCR in Fig. 3. Boxplots show the SCR scores for a verifier across all the generated problems; variation in plots arises from the 5 trained versions of the networks for each problem. For each box, the middle line represent the median, the box-bounds are the first and third quartiles, and the whiskers represent minimal and maximal values.

The plot for $\textsc {MNIST}_{ConvBig}$ on the left of Fig. 3 shows that **the GDVB benchmark with the MNIST**$_{ConvBig}$ **seed is able to identify considerable performance variation across verifiers**, with $\textsc {ERAN} _{DP}$ and Neurify accurately verifying a median of over 20 properties, the rest of the ERAN-variants verifying between 10 and 13 properties, and the remaining tools verifying between 0 and 8 properties. The results are consistent when we employ DAVE-2 as the seed network, with **marked differences among groups of verifiers** although the generated problems turned out to be more challenging across all verifiers. $\textsc {ERAN} _{DP}$ and Neurify, the top performers, can verify less than half of the generated problems. Verifiers like BaB were unable to verify any problem derived from DAVE-2 because of the complexity of the seed problem. This point highlights the need for benchmarks to evolve with networks that incorporate emerging technology, and also GDVB ’s ability to automatically generate a benchmark from different seeds to address that need.

**Fig. 4.**
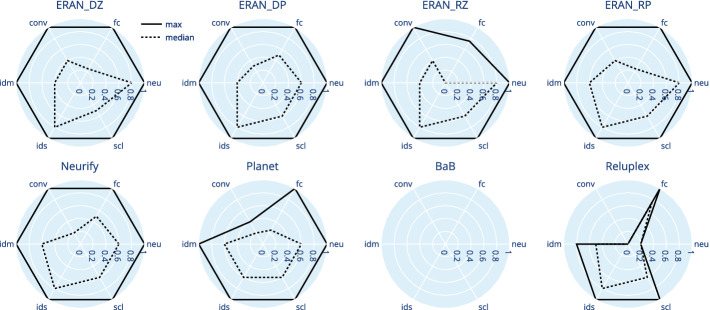
DAVE-2: radar plot with maximum (solid) and median (dotted) values

**Fig. 5.**
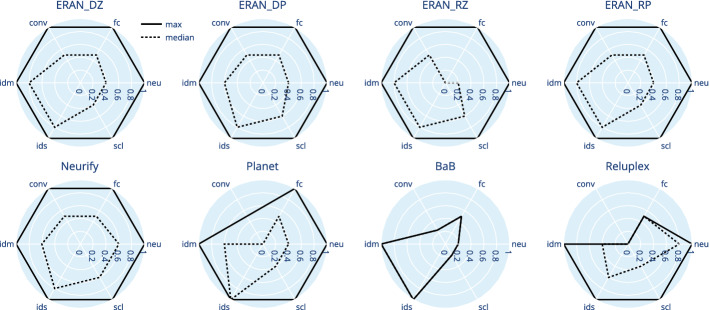
$\textsc {MNIST}_{ConvBig}$: radar plot with maximum (solid) and median (dotted) values

Now, understanding the overall performance of a family of verifiers is useful but it is likely just the first step for a researcher to understand under what conditions a verifier excels or struggles. When such conditions correspond to the factors manipulated by GDVB, then they are readily available for further analysis. One analysis may consist of simply plotting the data across its multiple dimensions. We do so in the form of radar-charts for DAVE-2 in Fig. 4 and for $\textsc {MNIST}_{ConvBig}$ in Fig. 5,^4^. Since the observations we can gather from both networks are similar, we just discuss DAVE-2 in detail. Each chart includes six axes representing a factor scaled between 0 and 1. The solid lines link the maximum values across factors that were accurately verified while the dotted lines link the median values across factors.

The shape of the lines in the radar plots clearly show that the **verification problems generated by** GDVB **reveal unique patterns across the verifiers**. For example, the ReLuplex plot indicates that it can do well verifying networks with multiple fully connected (fc) layers but is challenged by larger networks (neu) and those with convolutional layers (conv). Comparing multiple charts also reveals some interesting trade-offs. For example, for smaller networks with just fully connected layers, the medians seem to indicate that ReLuplex is better than Planet. However, when a network incorporates convolutional layers or a larger number of neurons, Planet appears to outperform ReLuplex.

Looking across charts can also pinpoint specific improvements resulting from tool extensions or revisions. For example, the median line of $\textsc {ERAN} _{RZ}$ indicates that it was not as effective in handling verification problems with a larger number of layers as its predecessor $\textsc {ERAN} _{DZ}$; the same trend holds for the pair $\textsc {ERAN} _{RP}$ and $\textsc {ERAN} _{DP}$. We note that a more restrictive benchmark that is biased towards fewer fully connected layers might not reveal such differences.

GDVB offers the opportunity to investigate such differences even further by generating targeted verification problems for a subset of factors hypothesized to be culprits of those differences. For example, GDVB could generate additional verification problems with a number of fully connected layers between 60% and 80% of the total, while keeping the other factors constant, to refine the understanding of the differences between $\textsc {ERAN} _{RZ}$ and $\textsc {ERAN} _{DZ}$.

This study illustrates how GDVB benchmarks support the exploration of verifier performance, lowering the burden on researchers to manually prepare tens to hundreds of verification problems, and reducing the opportunities for bias.

### GDVB and Benchmark Requirements R1–R3

As explained in Sect. 1, benchmarking in verification seeks to develop benchmarks that are: diverse; representative of real use cases; and reactive to new technologies. The previous sections have provided evidence of how, through its generative nature, GDVB is reactive to new advances in technology included in the seed network. We have also seen the high degree of parameterization GDVB offers including for setting a seed network from which realistic attributes are inherited in the generated verification problems. In this section we want to illustrate how GDVB addresses the diversity requirement.

**Fig. 6.**
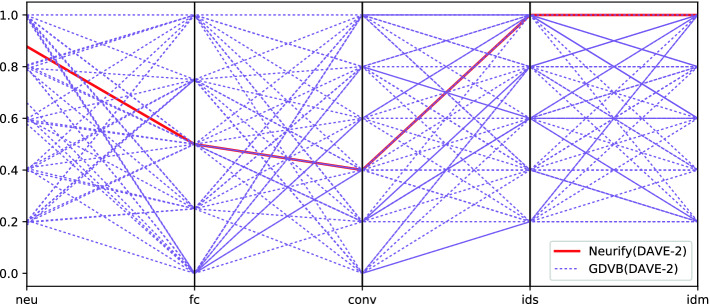
Diversity explored across factor levels

To depict diversity we use the parallel coordinate graph in Fig. 6. Each vertical line corresponds to a factor, and the markers in each vertical line corresponds to an explored level. Each verification problem is a polyline that connects the factors’ levels explored by it. The two sets of lines correspond to the verification problems included in the DAVE-2 benchmark published with Neurify [59], which is a downsized version of the full DAVE-2 DNN, and the benchmark produced by GDVB (DAVE-2, ...). Each factor in the plot is normalized by dividing by the maximum value for the factor.

Figure 6 shows that the Neurify ’s DAVE-2 has a large number of neurons, inputs, and dimensions. Yet, it provides very limited coverage of all the factor levels that may affect verification performance. In contrast, GDVB provides a systematic exploration of the factors levels that can affect verifier performance making it much less biased – especially to the numbers of layers in the verification problems, and the combination of those factor levels.

The parallel plot for GDVB benchmark with the $\textsc {MNIST}_{ConvBig}$ seed (not shown for space reasons), depicts a similar trend in terms of systematic exploration of diversity, but since $\textsc {MNIST}_{ConvBig}$ is simpler than DAVE-2, the generated benchmark is correspondingly simpler. This points to the need to identify representative and challenging seeds when parameterizing GDVB. GDVB is fully capable of accomodating factor levels that exceed 100% of a seed network, which is a means of pushing verifiers to the limits of their abilities.

We note that excluding factors or levels can yield a systematically generated benchmark that is unable to characterize differences between verifiers, or worse, misleads such a characterization by emphasizing certain factors while overlooking others. For example, not exploring different network sizes or exploring networks sizes under 1000 neurons will render similar scores across many DNN verifiers that are differentiated by more comprehensive benchmarks. In applying GDVB, we suggest selecting as many factors as we know may matter, starting from a challenging seed problem, and incrementally refining the levels as needed to focus benchmark results to differentiate verifier performance.

## Conclusion

The increasing adoption of DNNs has led to a surge in research on DNN verification techniques. Benchmarks to assess these emerging techniques, however, are costly to develop, often lack in diversity and do not represent the population of real evolving DNNs. To address this challenge, we have introduced GDVB, a framework for systematically generating DNN verification problems seeded in complex, real-world networks, ensuring that benchmarks are derived from real problems. GDVB is parameterizable by the factors that may influence verification performance and thereby supports scalable benchmarking. A preliminary study, using 9 DNN verifiers, demonstrates how GDVB can support the assessment of the state-of-the-art.

We plan to conduct broader studies of verifier performance using GDVB, and we encourate other researchers to use and contribute to it. There are many directions to explore in identifying new factors that influence performance, e.g., the impact of quantization and model compression approaches [26]. Work in this direction promises to deepen the community’s understanding and lead to advances in DNN verification.

## Footnotes

1 Since unsatisfiability requires the consideration of all possible variable assignments which generally is more costly than finding a single satisfiable assignment.

2 We cross-check accurate results with multiple verifiers.

3 https://github.com/edwardxu0/GDVB.

4 We do not plot BaBSB as its performance was identical to BaB.
